# *Fusobacterium nucleatum* Acts as a Pro-carcinogenic Bacterium in Colorectal Cancer: From Association to Causality

**DOI:** 10.3389/fcell.2021.710165

**Published:** 2021-08-20

**Authors:** Shuang Wang, Yang Liu, Jun Li, Lei Zhao, Wei Yan, Baiqiang Lin, Xiao Guo, Yunwei Wei

**Affiliations:** Department of Oncological and Endoscopic Surgery, The First Affiliated Hospital of Harbin Medical University, Harbin, China

**Keywords:** colorectal cancer, *Fusobacterium nucleatum*, tumor microenvironment, metastasis, chemoresistance

## Abstract

Colorectal cancer (CRC) is a common cancer worldwide with complex etiology. *Fusobacterium nucleatum* (*F. nucleatum*), an oral symbiotic bacterium, has been linked with CRC in the past decade. A series of gut microbiota studies show that CRC patients carry a high abundance of *F. nucleatum* in the tumor tissue and fecal, and etiological studies have clarified the role of *F. nucleatum* as a pro-carcinogenic bacterium in various stages of CRC. In this review, we summarize the biological characteristics of *F. nucleatum* and the epidemiological associations between *F. nucleatum* and CRC, and then highlight the mechanisms by which *F. nucleatum* participates in CRC progression, metastasis, and chemoresistance by affecting cancer cells or regulating the tumor microenvironment (TME). We also discuss the research gap in this field and give our perspective for future studies. These findings will pave the way for manipulating gut *F. nucleatum* to deal with CRC in the future.

## Introduction

Colorectal cancer (CRC), known for its high morbidity and mortality, is prevalent worldwide and increasing the global health burden (Keum and Giovannucci, 2019). In 2020, CRC was responsible for the second-highest number of cancer-related deaths in men and women (Siegel et al., 2020). Though it has shown a decline in overall morbidity and ranks third among all cancers in recent years, the number of affected individuals is rapidly shifting from elderly people to young and middle-aged people (Hofseth et al., 2020; Siegel et al., 2020; Akimoto et al., 2021). The causes of CRC are complex and varied, and the interaction between genetic background and environmental stimuli contributes to the development of CRC (Lichtenstein et al., 2000). It is estimated that genetic factors account for only 10–30% of the CRC risk; therefore, environmental factors may play a significant role in causing sporadic CRC (Lichtenstein et al., 2000; Jasperson et al., 2010; Jiao et al., 2014; Graff et al., 2017). A high fat and low fiber diet, smoking, heavy drinking, and physical inactivity all could increase the risk of CRC (Song et al., 2020; Yue et al., 2021). Gut microbiota, which mainly resides in the colon, has been recognized as an essential contributor to CRC in recent years.

The gut microbiota consisting of bacteria, archaea, viruses, fungi, protozoa, and helminths reflects a micro-ecosystem that intensely interacts with the host (Lynch and Pedersen, 2016). Physically, the microbiota performs many functions, such as fiber digestion and immune modulation (Adak and Khan, 2019). However, several factors, such as gut inflammation and changes in dietary habits, may alter the composition and function of gut microbiota, which is commonly termed dysbiosis (Weiss and Hennet, 2017; Illiano et al., 2020). This gut microbiota dysbiosis plays a role in intestinal and extraintestinal diseases, such as inflammatory bowel disease and cardiovascular diseases (Glassner et al., 2020; Jin et al., 2020). With the advancement of 16S rRNA and shotgun metagenomics, researchers have confirmed that CRC patients are accompanied by gut microbiota dysbiosis, characterized by an increase in cancer-associated bacteria such as *polyketide synthase Escherichia coli* (*pks^+^ E. coli*), enterotoxigenic *B. fragilis* (*ETBF*), *Fusobacterium nucleatum (F. nucleatum)* and others, whereas protective or beneficial bacteria such as *Clostridium Faecalibacterium* are reduced (Janney et al., 2020). While, *F. nucleatum* has attracted more attention in CRC studies (Castellarin et al., 2012; Kostic et al., 2012). In this review, we first introduce the biological characteristics of *F. nucleatum*, and then summarize the clinical findings of *F. nucleatum* in kinds of CRC patients’ samples. We also highlight its role as a pro-carcinogenic bacterium in various CRC models and the specific mechanisms. Finally, we put forward feasible insights for further studies in *F. nucleatum* and CRC.

## The Biological Characteristics of *F. nucleatum*

*Fusobacterium nucleatum* is a fusiform or spindle-shaped gram-negative anaerobe that mainly colonizes the oral mucosa. As a symbiotic bacterium, *F. nucleatum* serves as a structural support for other bacteria to form the oral biofilms, which are essential for the normal oral microenvironment (Lamont et al., 2018; Zhang et al., 2018). On the other hand, since it has been isolated from clinical infections and multiple tumor samples, such as periodontitis (Kim E. H. et al., 2020), adverse pregnancy (Vander Haar et al., 2018; Figuero et al., 2020), appendicitis (Hattori et al., 2019), CRC (Castellarin et al., 2012; Kostic et al., 2012), and breast cancer (Parhi et al., 2020), it has been regarded as an opportunistic pathogen and a tumor-associated bacterium. To further explore its mechanisms to promote CRC, we first introduce four basic biological characteristics associated with pathogenicity.

First and foremost, *F. nucleatum* is an adhesive bacterium that expresses multiple adhesins on its surface, including RadD, Aid1, and FomA, which can mediate the coaggregation of bacteria to form biofilms (Liu et al., 2010; Kaplan et al., 2014; Guo et al., 2017). Not only in the oral cavity (Bowen et al., 2018), but also in CRC, bacterial biofilms composed of *F. nucleatum* are widely present, especially in right-sided colon cancer (Dejea et al., 2014; Drewes et al., 2017; Li et al., 2017).

Second, *F. nucleatum* can adhere to and even invade various host cells, including epithelial cells, endothelial cells, and fibroblasts (Han, 2015). The downstream responses of host cells induced by adherence and invasion are the primary conditions for *F. nucleatum* to exert its virulence and pathogenicity. At present, the well-known virulence factors/adhesins that connect *F. nucleatum* to host cells are as follows. (1) FadA, a complex composed of immature pre-FadA and surface mature mFadA in different proportions, is the specific and best-characterized virulence factor of *F. nucleatum* (Xu et al., 2007). The invasion of *F. nucleatum* is mainly attributed to FadA, and once FadA binds to the corresponding ligands, it can activate the zipper mechanism to transport *F. nucleatum* into cells (Fardini et al., 2011; Rubinstein Mara et al., 2013). (2) Fap2, the largest outer membrane protein of *F. nucleatum*, is a galactose-inhibitable adhesin (Coppenhagen-Glazer et al., 2015). It is the primary molecule that regulates *F. nucleatum* adherence to various cancer cells, such as colorectal and breast cancer cells, which usually overexpress its specific ligand, Gal-GalNAc (Abed et al., 2016; Parhi et al., 2020).

Third, *F. nucleatum* has vast metabolic potential. On the one hand, *F. nucleatum* has multiple enzymes that produce hydrogen sulfide (H_2_S) from L-cysteine (Basic et al., 2017; Kezuka et al., 2018; Blachier et al., 2019). H_2_S, which is the cause of oral malodor (Hampelska et al., 2020), can stimulate cell proliferation in CRC (Szabo et al., 2013). On the other hand, *F. nucleatum* is identified as a potential butyrate producer (Vital et al., 2014; Llama-Palacios et al., 2020; Dahlstrand Rudin et al., 2021). Unlike other bacteria, it does not rely on polysaccharides to produce butyrate but uses amino acids (Vital et al., 2014). The role of butyrate in CRC has always been the focus of controversy (Guan et al., 2021), and whether butyrate from *F. nucleatum* plays a role in the CRC-associated microenvironment is still unknown. Furthermore, its outer membrane contains lipopolysaccharide (LPS), which can play a vital role in causing inflammation or tumorigenesis through LPS receptors, such as Toll-like receptor 4 (TLR4) (Chen et al., 2017).

Fourth, like other gram-negative bacteria, *F. nucleatum* can release extracellular vesicles (EVs) or outer membrane vesicles (OMVs) (Liu et al., 2019; Liu L. et al., 2021), which contain a large number of bioactive substances and participate in bacteria-bacteria or bacteria-host cells communications (Macia et al., 2019). By isolating and purifying *F. nucleatum* (ATCC 23726)-derived EVs, researchers verified that EVs’ surface protein, FomA, could bind to TLR2 on the intestinal epithelial cells and then modulate innate immunity (Martin-Gallausiaux et al., 2020). *F. nucleatum* EVs’ can also disrupt the intestinal epithelial barrier by activating the FADD-RIPK1-caspase3 signaling pathway to promote necrosis of intestinal epithelial cells (Liu L. et al., 2021). Furthermore, Engevik et al. (2021) used OMVs isolated from *F. nucleatum* (ATCC 10953) to stimulate colonic epithelial cells and found the production of the proinflammatory cytokines IL-8 and TNF increased. These studies suggest that EVs or OMVs are also a form of pathogenic effect.

## *F. nucleatum* and CRC: Clinical Studies in Different Sample Types

Before exploring the associations between *F. nucleatum* and CRC, it is necessary to determine the existence and abundance of *F. nucleatum* in different sample types. There are numerous qualitative and quantitative methods for studying *F. nucleatum*, including whole-genome shotgun sequencing, quantitative PCR (qPCR), 16S rRNA sequence analysis, fluorescence *in situ* hybridization (FISH), RNA sequencing, and bacterial culture. Moreover, with the development of technology, droplet digital PCR (ddPCR) and fluorescence quantitative PCR (FQ-PCR) have gradually become new quantitative methods. Among these methods, bacterial culture and FISH provide the strongest evidence, as they can identify the presence of viable *F. nucleatum* and its position, respectively (Table 1).

**TABLE 1:** The advantages, clinical significance, and challenges of different sample types for *F. nucleatum* studies.

Sample type	*F. nucleatum* study method	Advantage and clinical significance	Challenge
Tissue samples	Whole-genome shotgun sequencing, 16S rRNA gene sequencing, RNA-sequencing, qPCR, FQ-PCR, ddPCR, FISH, isolate cultured	1. Represent the microbiota which is equipped the closest relationship with CRC. 2. Intratumoral *F. nucleatum* can act as a prognostic and predictive biomarker for CRC patients.	*F. nucleatum* studies should take into account tumor heterogeneity, as the features of *F. nucleatum* may vary according to distinct tumor locations.
Mucosal samples	16S rRNA gene sequencing, FISH	1. Mucosal samples can be collected with rectal swabs, and allow flexible time-point sampling for studies with specific purposes. 2. Provide more information on colon biofilms, which include symbolic and antagonistic bacteria for *F. nucleatum*. 3. Can be collected from normal people to establish healthy controls for clinical studies following legal/ethical guidelines.	1. Sensitive to clinical intervention, such as bowel preparation, so the results should be interpreted carefully. 2. When a mucosal biopsy sample is obtained, there is inevitable cross-contamination, such as that from the intestinal fluid from a non-fixed point.
Fecal samples	16S rRNA gene sequencing, qPCR, ddPCR, metagenomics	1. This method is easy and noninvasive for sample collection and an ideal sampling method for practical clinical applications. 2. Give an overview of the whole gut microbiota and metabolome, provide information on bacterial-associated metabolites, and identify the relationship between the gut micro-ecosystem and CRC. 3. Fecal *F. nucleatum* alone or in combination with other tests can be used as a noninvasive screening biomarker for CRC.	1. Can be affected by diet, bowel habits, and health conditions. 2. Cannot accurately reflect the microbiota at the tumor site.
Serum and oral samples	ELISA, multiplex serology 16S rRNA gene sequencing, isolate cultured	1. Easy accessibility, available for multiple time-point studies. 2. Oral microbiota can act as a noninvasive and convenient biomarker for detecting CRC.	1. The sensitivity and specificity of *F. nucleatum*-specific antibodies in the serum for the diagnosis of CRC is poor. 2. The precise interaction between oral-gut *F. nucleatum* has not been yet identified.

*qPCR, quantitative real-time PCR; FISH, fluorescence *in situ* hybridization; FQ-PCR, fluorescence quantitative PCR; ddPCR, droplet digital.*

### Tissue Samples

Considering that microbiota in direct contact with epithelial cells is likely to influence the progression of CRC, microbiome analysis of tissue samples can provide highly useful data. The tumor tissue samples examined include postoperative fresh frozen tissues (Castellarin et al., 2012; Kostic et al., 2012; Li et al., 2016; Wei et al., 2016; Ye et al., 2017; Yamaoka et al., 2018), formalin-fixed paraffin-embedded (FFPE) tissue blocks (Ito et al., 2015; Mima et al., 2015, 2016a,b; Park et al., 2017; Liu et al., 2018), and patient-derived tumor xenografts (PDXs) (Bullman et al., 2017). Castellarin et al. (2012) and Kostic et al. (2012) first suggested the existence of intratumoral *F. nucleatum*, and this conclusion was fully confirmed in multiple samples and cohorts. At the level of subcellular localization, FISH revealed that *F. nucleatum* was located close to the lumen and ulcerated regions, predominantly associated with malignant cells (Kostic et al., 2012; Bullman et al., 2017). Transmission electron microscopy even showed that *F. nucleatum* invaded cancer cells and existed in a vesicle structure (Bullman et al., 2017). As a result, intratumoral *F. nucleatum* is considered invasive. Except for CRC tissues, *F. nucleatum* can be detected in secondary distal metastases such as liver (Bullman et al., 2017), lung (Abed et al., 2016), and lymph node (Abed et al., 2016) as well as in PDX models (Bullman et al., 2017).

The abundance of *F. nucleatum* in *F. nucleatum*-positive CRC is based on the following characteristics. (1) *F. nucleatum* is significantly enriched in tumor tissues compared to adjacent normal tissues, even a mean 415 times higher (Castellarin et al., 2012). (2) The abundance of *F. nucleatum* varies and increases along with the adenoma-carcinoma sequence (Flanagan et al., 2014; Nakatsu et al., 2015). (3) Patients with metastasis or recurrence have more intratumoral *F. nucleatum* than patients without metastasis or recurrence (Chen Y. et al., 2020; Yu et al., 2017). (4) Even in the same tumor tissues, the abundance of *F. nucleatum* varies at different sampling sites (Liu W. et al., 2021).

Based on the high amount of intratumoral *F. nucleatum* and the clinical, pathological, and molecular features of CRC, association studies revealed critical roles for *F. nucleatum*. (1) Several studies revealed that patients with higher intratumoral *F. nucleatum* usually had shorter survival times and were more likely to experience recurrence, which suggested using *F. nucleatum* as a prognostic and predictive biomarker (Flanagan et al., 2014; Mima et al., 2016b; Yu et al., 2017). (2) Pathological connections seem to suggest that *F. nucleatum* participates in the occurrence and development of CRC, as *F. nucleatum* is related to different tumor phenotypes, such as autophagy and glycolysis (Yu et al., 2017; Hong et al., 2020). (3) Regarding its relationship with molecular features, intratumoral *F. nucleatum* is associated with high microsatellite instability, the CpG island methylator phenotype (CIMP) status, and certain gene mutations (Tahara et al., 2014). In addition to the cancer cells, *F. nucleatum* is also associated with low CD3^+^ T cells (Mima et al., 2015; Borowsky et al., 2021).

### Mucosal Samples

Mucosal samples examined include rectal swab samples (Chen et al., 2012), colonoscopy mucosal biopsy samples (Nakatsu et al., 2015; Tunsjo et al., 2019), and postoperative tissue samples with the mucus layer preserved (Dejea et al., 2014). With the methods mentioned at the beginning of the paragraph, *F. nucleatum* can be detected in all three mucosal samples of CRC patients, and its abundance is higher than that in healthy controls. Mucosal samples contain the microbiota in the superficial layer of the tissue, which is called “mucosa-adherent microbiota” (Chen et al., 2012). To emphasize their association with tumor tissues, the mucosa-adherent microbiota, and tissue microbiota are collectively called the “mucosa-associated microbiota” (Chen et al., 2012). Rectal swabs come in close contact with feces, so their microbiota structure is similar to that in feces in some degree (Chen et al., 2012). However, rectal swabs can allow flexible time-point sampling for studies with specific purposes. As for biopsy samples, although the sampling area is slightly small, the sampling process is inevitably contaminated, and bowel preparation before inspection may destroy the microbiota (Tang et al., 2020), they have the advantage of allowing the inclusion of healthy controls, which is a limitation of clinical studies because the collection of tissue samples is restricted by ethical requirements. Tissue samples with a preserved mucus layer are mainly used to study colon biofilms, which are essential to understand the impacts of multimicrobiota interactions on CRC (Drewes et al., 2017).

### Fecal Samples

Although the microbiota in the feces is easily affected by diet, bowel habits, and other factors (Falony et al., 2018), cohort studies in multiple countries have confirmed that both CRC and advanced adenoma patients have an increased abundance of *F. nucleatum* in feces (Wu et al., 2013; Yu et al., 2015; Eklof et al., 2017; Suehiro et al., 2017; Wong et al., 2017a; Guo et al., 2018), although there are some studies that cannot confirm this (Amitay et al., 2017). Fecal samples usually represent gut luminal microbiota, but the structure of the microbiota differs between various lumen locations (Tanca et al., 2017). Therefore, fecal samples cannot truly reflect the luminal microbiota corresponding to tumor tissues (Flanagan et al., 2014), which is a great limitation to study their direct pathogenic effects on CRC. However, fecal samples not only provide an overview of the whole gut microbiota changes, but also provide information on bacterial-associated metabolites, and reveal the relationship between the gut micro-ecosystem and CRC (Kim et al., 2020). Moreover, their easy accessibility makes them better screening biomarkers than tissue or mucosal samples. Studies have shown that the combination of the fecal immunochemical test (FIT) and quantitation of fecal *F. nucleatum* or the ratio of *F. nucleatum* to *Faecalibacterium prausnitzii* can improve the screening sensitivity and specificity of advanced adenoma and CRC (Wong et al., 2017a; Guo et al., 2018). Moreover, the fecal *F. nucleatum* test may be an effective non-invasive predictor for metachronous adenoma after endoscopic polypectomy (Xue et al., 2021).

### Serum and Oral Samples

Since *F. nucleatum* infection can stimulate the body to produce specific antibodies, several researchers used ELISA and multiplex serology to detect *F. nucleatum*-specific IgA and IgG antibodies in the serum and then evaluated their sensitivity and specificity to diagnose CRC. However, the results were disappointing. Kurt and Yumuk (2021) revealed that the diagnostic accuracy of IgA and IgG was poor, and Wang et al. (2016) suggested that only combining IgA, CEA, and CA199 could improve the diagnostic sensitivity. Furthermore, numerous diseases in addition to CRC can cause an increase in antibodies of *F. nucleatum* in the serum (Yamamura et al., 2017), so the results should be interpreted carefully. However, in a prospective cohort study, researchers found that serum *F. nucleatum* antibody level was not associated with CRC incidence, which indicated that *F. nucleatum* infection did not increase the risk of CRC (Butt et al., 2019; Lo et al., 2021). In contrast to the cross-sectional clinical studies, this study gives some hints for the cause-effect relationship between *F. nucleatum* and CRC.

Oral samples examined mainly include saliva, oral wash, tongue coating, and oral swabs (Zhang et al., 2020). The oral microbiota has been associated with several cancers, such as pancreatic cancer (Fan et al., 2018), esophageal cancer (Peters et al., 2017), and CRC (Han, 2014). Flemer et al. (2018) found that the oral swab microbiota of CRC patients was significantly different from that of healthy controls. Using the oral swab microbiota alone or in combination with the fecal microbiota can distinguish CRC patients from healthy controls, so the oral microbiota may act as a non-invasive biomarker for detecting CRC (Flemer et al., 2018). Zhang et al. (2020) reached a similar conclusion in a Chinese cohort. However, Flemer et al. (2018) detected the abundance of *Fusobacterium* in both oral and CRC tissues, finally found that there was no association between them in quantity.

In summary, the abundance of *F. nucleatum* in tissue, mucosal biopsy, and fecal samples of CRC is higher. Considering that each kind of sample has specific characteristics and scientific investigation values, researchers should apply the appropriate sampling methods according to the aim of the study (Table 1).

## The Source, Translocation, and Colonization of *F. nucleatum*

As *F. nucleatum* is rarely found in the healthy gut, researchers have tried to explore the origin of *F. nucleatum* and how it migrates to and colonizes CRC tissues. For this purpose, Flemer et al. (2018) analyzed the microbiota in oral swabs, colonic mucosa, and feces, and ultimately found a similar network between colonic mucosal bacteria and oral bacteria. Komiya et al. (2019) and Abed et al. (2020) studied this problem in-depth and suggested that gut *F. nucleatum* originated from the oral cavity. Not only was the oral cavity the main habitat of *F. nucleatum*, but identical strains of *F. nucleatum* existed in CRC tissues and saliva specimens obtained from the same patient. However, several studies have shown that the virulence of *F. nucleatum* varies from the oral cavity to the gut, and the latter seems to be more virulent (Chen et al., 2018).

There are two possible routes for *F. nucleatum* to translocate from the oral cavity to the gut. Abed et al. (2016) injected *F. nucleatum* via the tail vein into orthotopic CRC mouse models and found *F. nucleatum* in tumors, which verified that blood-borne *F. nucleatum* could localize to CRC tissues. This is consistent with the clinical phenomenon that patients with *F. nucleatum* bacteremia were at an increased risk of CRC (Kwong et al., 2018). Another way is by swallowing through the digestive tract. Kostic et al. (2013) gave *F. nucleatum* to *Apc ^Min/+^* mice by gavage every day and harvested more and larger tumors from these mice than control mice. Simultaneously, they found *F. nucleatum* in the tumor tissues by qPCR and FISH. By comparing these two routes, Abed et al. (2020) were more inclined to support the blood-borne route because compared to oral *F. nucleatum*, intravenous *F. nucleatum* was more effective at colonizing tumors. Furthermore, blood-borne *F. nucleatum* can avoid the attack of the digestive tract. However, although previous studies showed that *F. nucleatum* was present in liver and lymph node metastases (even viable *F. nucleatum* in liver metastases) (Abed et al., 2016; Bullman et al., 2017), the mechanisms that mediate the transport of *F. nucleatum* to distal metastases are still unclear. One possible mechanism is that *F. nucleatum* invades cancer cells and reaches the liver or lymph nodes by blood circulation or lymphatic circulation. Alternatively, during CRC, *F. nucleatum* may migrate from the oral cavity to the liver through the blood circulation and participate in the development of liver metastases.

Regarding colonization, CRC is an essential precondition for *F. nucleatum* colonization. Abed et al. (2016) unveiled that adhesin Fap2 bound to Gal-GalNAc, a sugar residue overexpressed on the surface of CRC cells, and then mediated the attachment of *F. nucleatum*. Moreover, in metastatic lesions, such as liver and omentum, Gal-GalNAc is also overexpressed, which suggests that *F. nucleatum* colonization here depends on Fap2-Gal-GalNAc as well. *F. nucleatum* strains with Fap2 absent or mutated can reduce this colonization. The combination of adhesin FadA with E-cadherin, which presents on the surface of CRC cells, can also promote colonization (Rubinstein Mara et al., 2013). However, unlike Gal-GalNAc, since E-cadherin is not specifically overexpressed on cancer cells, the attachment of *F. nucleatum* may be largely attributed to Fap2-Gal-GalNAc.

## *Fusobacterium nucleatum* Is a Putatively Pro-Carcinogenic Bacterium in CRC Models

During the initial exploration of the gut microbiota in CRC, researchers transplanted fecal or biofilm samples into mice and successfully accelerated tumorigenesis (Wong et al., 2017b; Tomkovich et al., 2019). Tumorigenic mechanisms, such as inflammation, immune regulation, genotoxins, and harmful metabolites, have been completely reviewed in other studies (Janney et al., 2020). However, fecal or biofilm samples include not only a variety of microbiota but also multiple metabolites. As such, there are great limitations to precisely manipulate fecal or biofilm samples, and using antibiotics to abolish the whole microbiota will cause unpredictable effects. It is urgent to identify the specific microbiota that plays a key role in tumorigenesis.

Since many studies showed that *F. nucleatum* was enriched in the tumor tissue and even *F. nucleatum* can invade into the tumor cell of CRC, it is reasonable to suspect *F. nucleatum* could influence tumorigenesis. Therefore, many studies attempted to prove the tumorigenic ability of *F. nucleatum* with various experimental models. These models are roughly classified into three categories, *in vitro* co-culture system, 3D intestinal organoids, and CRC mouse models. The co-culture with cancer cells *in vitro* is the simplest and classical way to study carcinogenic changes, but there are still some limitations to applying the results to the *in vivo* environment. The organoid is a model which plays an important role in stimulating the gastrointestinal environment *in vitro* (Drost and Clevers, 2018). The application of the organoid model not only proved that the *pks^+^ E. coli* could cause the formation of CRC (Pleguezuelos-Manzano et al., 2020), but also proved that certain substances secreted by *F. nucleatum* could stimulate organoids to secrete TNF and increase intestinal inflammation (Engevik et al., 2021). Considering that the native host physiological condition is a critical feature for a successful tumor model, the mouse model is still the ideal model for cancer studies, so we summarized the mouse models that were used in the studies on *F. nucleatum* and CRC progression. In addition, since *F. nucleatum* has been found to exist in metastatic and recurrent CRC tissues, its effects on promoting tumor metastasis and inducing chemoresistance have also been investigated (Table 2).

**TABLE 2:** Etiological studies of *F. nucleatum* in CRC mouse models.

CRC stage	*F. nucleatum* strain	CRC mouse models	*F. nucleatum* administration route	Findings/mechanisms	References
Tumor progression	ATCC 12230 (FadAc, mFadA, US1)	Nude mice HCT116 subcutaneously	Intratumor injection	Induces oncogenic and inflammatory responses via FadA	Rubinstein Mara et al. (2013)
		APC *^*Min*+^* mice	Oral gavage	Activates the Wnt/β-catenin oncogenic pathway via FadA	Rubinstein et al. (2019)
	ATCC 25586 (US1)	Nude mice HCT116 subcutaneously	Intratumor injection	Promotes high glucose metabolism to provide energy for tumorigenesis	Hong et al. (2020)
		Nude mice HT29/HCT116/Lovo subcutaneously	Co-cultured	Causes DNA damage via FadA Induces miR-21 to activate the MAPK oncogenic pathway	Yang et al. (2017); Guo P. et al., 2020; Guo P. et al., 2020
		APC*^*Min*+^* mice	Oral gavage	Causes DNA damage via FadA Induces miR-21 to activate the MAPK oncogenic pathway Activates the JAK/STAT and MAPK/ERK pathways	Yu et al. (2015); Yang et al. (2017); Guo et al., 2020
		WT mice AOM/DSS	Oral gavage	Induces miR-21 to activate the MAPK oncogenic pathway	Yang et al. (2017)
		WT mice DMH	Oral gavage	Activates the JAK/STAT and MAPK/ERK pathways	Yu et al. (2015)
	F01	APC *^*Min*+^* mice	Oral gavage	Promotes M2 polarization of macrophages Via a TLR4/p-PAK1/p-β-catenin S675 cascade	Chen et al. (2018); Wu et al. (2018)
	COCA36F3	Human-derived xenografts	/	Metronidazole reduces *F. nucleatum* load and tumor growth	Bullman et al. (2017)
	EAVG_002	APC *^*Min*+^* mice	Oral gavage	Modulates the tumor-infiltrating immune cells	Kostic et al. (2013)
		IL-10^–/–^ mice	Oral gavage	No findings	Kostic et al. (2013)
		T-bet^–/–^× Rag2^–/–^ mice	Oral gavage	No findings	Kostic et al. (2013)
	CC53, CC7/3JVN3C1, CC7/5JVN1A4, CC2/3Fmu1, CC2/3FmuA, CC7/4Fmu3	APC *^*Min*+^*; IL-10^–/–^ mice (GF)	Oral gavage	No findings	Tomkovich et al. (2017)
		APC *^*Min*+^* mice (GF)	Oral gavage	No findings	Tomkovich et al. (2017)
	CC7/4Fmu3	APC *^*Min*+^* mice (GF)	Oral gavage	No findings	Tomkovich et al. (2017)
Tumor metastasis	ATCC 25586	Nude mice HCT116 tail vein injection	Co-cultured	Modulates KRT7-AS/KRT7 to promote metastasis	Chen S. et al., 2020
		Nude mice HCT116 subcutaneously	Exosomes* Intratumor injection	Stimulates cancer cells to generate miR-1246/92b-3p/27a-3p-rich exosomes to promote non-infected cancer cells migration	Guo S. et al., 2020
		Nude mice CT26 tail vein injection	Exosomes* Tail vein injection	Stimulates cancer cells to generate miR-1246/92b-3p/27a-3p-rich exosomes to promote non-infected cancer cells migration	Guo S. et al., 2020
	F01	WT mice CT26 tail vein injection	Co-cultured	Activates cancer-related autophagy by upregulating CARD3 expression	Chen Y. et al. (2020)
Chemoresistance	ATCC 25586	Nude mice HCT116/SW480 Subcutaneously	Intratumor injection	Downregulates miR-18a* and miR-4802 then activates the autophagy pathway Upregulates BIRC3 gene via TLR4/NF-κB pathway then inhibits apoptosis	Yu et al. (2017); Zhang et al. (2019)
Translocation and colonization	ATCC 23726 (K50, D22, CTI-2, CTI-7)	WT mice Orthotopic rectal CT26 intravenous injection	Tail vein injection	Translocates to CRC tissue by blood-borne The colonization is Fap2 dependent	Abed et al. (2016)
		APC *^*Min*+^* mice	Tail vein injection	The colonization is Fap2 dependent	Abed et al. (2016)

*Exosomes, isolated from the supernatant of *F. nucleatum*-infected HCT116 cells.

*WT, wild-type; AOM, azoxymethane; DSS, dextran sodium sulfate; DMH, 1,2-dimethylhydrazine; GF, germ-free.*

It needs to be mentioned that, though *F. nucleatum* has the potential to influence the progression, metastasis, and chemoresistance of CRC, its role in CRC initiation still needs to be further investigated. On the one hand, in some models, such as colitis-associated mice and germ-free *APC ^Min+^* mice, *F. nucleatum* intervention did not cause or accelerate tumorigenesis (Kostic et al., 2013; Tomkovich et al., 2017). On the other hand, even though clinical observations have found that the abundance of *F. nucleatum* was enriched up-raise in precancerous lesions before carcinogenesis, but this temporal precedence is only one of the criteria that are essential to build up the cause-effect relationship between *F. nucleatum* and CRC. Besides, unlike other well-known CRC tumor-causing bacteria, one clinical prospective cohort study found that serum *F. nucleatum* antibodies do not relate to CRC incidence rate (Butt et al., 2019, 2021; Lo et al., 2021). All these results indicate that *F. nucleatum* may not have an effect on CRC initiation, but further studies are needed to give a clear conclusion.

## The Mechanisms by Which *F. nucleatum* Is Involved in Various CRC Stages

**FIGURE 1:**
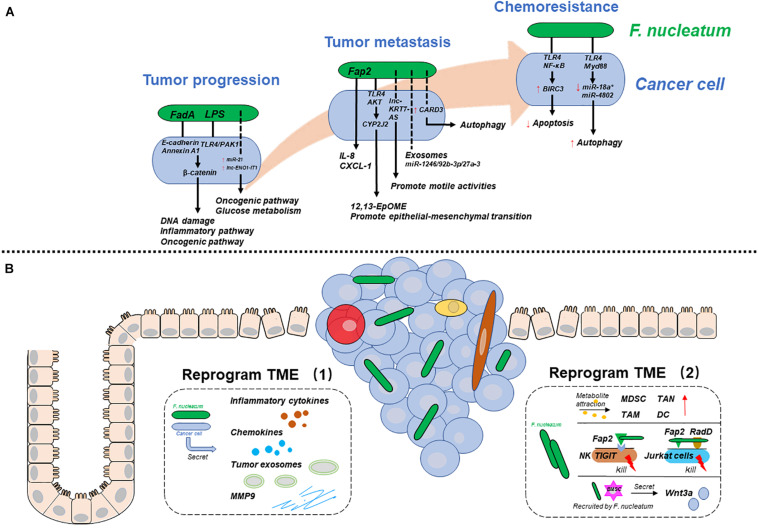
The mechanisms by which *F. nucleatum* is involved in various CRC stages. **(A)** *F. nucleatum* reprograms cancer cells during tumor progression, metastasis, and chemoresistance. **(B)** *F. nucleatum* reprograms the tumor microenvironment (TME) by interacting with cancer cells (left), immune cells and others (right). Solid lines represent proven mechanisms, and dotted lines represent unclear mechanisms.

Researchers have provided multiple mechanisms by which *F. nucleatum* participates in CRC progression, metastasis, and chemoresistance. In essence, *F. nucleatum* may influence CRC development by affecting cancer cells or their tumor microenvironment (TME) in different ways. Therefore, to clarify the mechanisms systematically, we will discuss two aspects: one is the mechanisms by which *F. nucleatum* reprograms cancer cells, and the other is the mechanisms by which *F. nucleatum* reprograms the TME to play a pro-carcinogenic role (Figure 1).

### *Fusobacterium nucleatum* Reprograms Cancer Cells

#### Tumor Progression

##### Focus on FadA

In addition to mediating adhesion and invasion, FadA also plays a key role in cellular oncogenesis (Rubinstein Mara et al., 2013; Rubinstein et al., 2019; Guo P. et al., 2020). Briefly, the binding of FadA and E-cadherin promotes the phosphorylation and internalization of the latter. At the same time, downstream β-catenin phosphorylation is reduced, and excess β-catenin accumulates in the cytoplasm and then translocates into the nucleus. Rubinstein Mara et al. (2013) verified that once the FadA/E-cadherin/β-catenin pathway was activated, the expression of inflammatory genes (such as NF-κB) and oncogenes (such as Myc and Cyclin D1) was increased. However, though both inflammatory genes and oncogenes were regulated by FadA/E-cadherin/β-catenin, activation of the former did not depend on the internalization of E-cadherin, while the latter required both its phosphorylation and internalization. Guo P. et al. (2020) also showed that *F. nucleatum* increased DNA damage and accelerated tumor growth through the FadA/E-cadherin/β-catenin pathway. This active pathway causes the overexpression of chk2, which is a multifunctional enzyme related to DNA damage. As a result, chk2 upregulation facilitates DNA damage and tumor growth. This is not the first study to find that *F. nucleatum* can affect DNA damage. Geng et al. (2020) reported that *F. nucleatum* induced DNA double-strand breaks in oral cancer cells, but there was no in-depth discussion on which factors of *F. nucleatum* cause this phenomenon. However, as E-cadherin is expressed on nearly all epithelial cells, it is difficult to attribute oncogenesis to FadA/E-cadherin. *In vitro* experiments showed that FadA bound to noncancerous cells expressing E-cadherin and promoted the expression of inflammatory genes, but it did not stimulate tumor growth (Rubinstein et al., 2019). This oncogenesis is specific to cancerous cells. Rubinstein et al. (2019) reported that in contrast to noncancerous cells, there was a special membrane protein called Annexin A1 on the surface of cancerous cells. Previous studies have verified that Annexin A1 is associated with the activation of Cyclin D1 and can stimulate CRC cell proliferation (Foo et al., 2019). To explore the relationship between FadA and Annexin A1, researchers used confocal microscopy and found that FadA, E-cadherin, Annexin A1, and β-catenin formed a complex on the surface of cancerous cells. There is a functional interaction among the components of the complex. On the one hand, Annexin A1 enhances the efficiency of FadA adhesion and invasion. On the other hand, FadA and E-cadherin increase Annexin A1 expression in CRC cells. Finally, through E-cadherin and Annexin A1, FadA causes β-catenin to enter the nucleus, which activates the expression of inflammatory genes and oncogenes, such as Cyclin D1, and then accelerates cell proliferation and tumor growth. Therefore, Annexin A1 is a critical component of FadA that has a stimulatory effect (Rubinstein et al., 2019). Approximately 90% of CRCs are associated with the activation of β-catenin signaling, and *F. nucleatum* is the main activator (Matly et al., 2021). Not only FadA but also the LPS produced by *F. nucleatum* can activate β-catenin signaling. Chen et al. (2017) reported that *F. nucleatum* and LPS induced β-catenin nuclear accumulation in CRC cells via the TLR4/PAK1 pathway. Although it is theoretically believed that the activation of β-catenin can promote cell proliferation, this study did not report whether LPS treatment alone could accelerate tumor growth. Therefore, the function of LPS alone is still unclear.

##### Focus on ncRNAs

Noncoding RNAs (ncRNAs), such as microRNAs and long ncRNAs, are crucial regulators of epigenetic genes, and their expression is dysregulated in cancer cells (Dragomir et al., 2020). Recent studies based on pathogenic bacteria have revealed that ncRNAs are a crosstalk factor between bacteria and host cells (Aguilar et al., 2019; Dong et al., 2019). Therefore, *F. nucleatum* may promote CRC by regulating gene expression through ncRNAs. Yang et al. (2017) found that the high expression of miR-21 in CRC was specifically induced by *F. nucleatum*. miR-21 reduces the expression of RAS1, which activates the MAPK pathway and enhances cell proliferation. Moreover, TLR4/Myd88/NF-κB may be an upstream pathway connecting *F. nucleatum* and miR-21. Although other bacteria such as *E. coli* can also activate TLR4/Myd88/NF-κB in cancer cells, they do not upregulate the expression of miR-21. This finding suggests that miR-21 is a *F. nucleatum*-specific oncogenic ncRNA. Hong et al. (2020) demonstrated that *F. nucleatum* enhanced abnormal glycolysis in cancer cells, which can provide more energy to promote cell proliferation and tumor growth. Mechanistically, *F. nucleatum* induces the transcriptional activation of the lncRNA ENO1-IT1, which binds to and interacts with KAT7, a subunit of the histone acetyltransferase complexes. The ENO1-IT1/KAT7 complex increases its target gene transcription by promoting histone acetylation. One of the target genes, ENO1, a key component of the glycolysis pathway, is transactivated and then alters the metabolic pathway of cancer cells.

#### Tumor Metastasis

##### Focus on cytokines

Cancer cells secrete cytokines during bacterial infections. Some cytokines affect cell proliferation and migration through autocrine mechanisms, and some act on adjacent cancer cells or noncancerous cells through paracrine mechanisms, which also play a crucial role in the development of tumors (Briukhovetska et al., 2021). Casasanta et al. (2020) treated HCT116 cells with *F. nucleatum* and detected cytokines in the culture medium. *F. nucleatum* induced the secretion of IL-8 and CXCL1, which are proinflammatory and prometastatic cytokines. Medium rich in IL-8 and CXCL1 enhanced the migration of uninfected cancer cells in a paracrine manner. However, the deletion of adhesin Fap2 caused *F. nucleatum* to lose the above functions. This research showed that Fap2 might be indispensable for the production of cytokines to enhance the migration ability of cancer cells.

##### Focus on ncRNAs

Noncoding RNAs may be another type of molecule through which *F. nucleatum* promotes migration and metastasis. Chen S. et al. (2020) performed RNA sequencing to detect the expression of ncRNAs in LoVo cells after incubation with *F. nucleatum*. Among the variable ncRNAs, the expression of the lncRNA KRT7-AS increased significantly, as did its target gene KRT7. Intracellular KRT7-AS promoted cell migration by enhancing the stability of KRT7. However, when KRT7-AS was depleted, the metastatic effect induced by *F. nucleatum* was abolished, which suggested that *F. nucleatum* promoted migration by upregulating KRT7-AS and KRT7. Mechanistically, *F. nucleatum* infection can activate the NF-κB signaling pathway, which acts as a transcription factor in the nucleus and promotes the transcription of KRT7-AS. In addition to playing a role intracellularly, ncRNAs, which are delivered by cancer cells then loaded into exosomes, also play a role in metastasis. Guo S. et al. (2020) revealed that *F. nucleatum*-infected associated tumor exosomes were larger than control exosomes and rich in ncRNAs. Among the ncRNAs, miR-1246/92b-3p/27a-3 expression was significantly increased and was absorbed by adjacent uninfected cancer cells. miR-1246/92b-3p/27a-3 can target GSK3β and activate the Wnt/β-catenin pathway to promote CRC cell migration *in vitro*. In summary, exosomes may magnify the effect of *F. nucleatum* on cancer cells. This conclusion, i.e., that *F. nucleatum*-infected exosomes facilitate CRC metastasis to intrahepatic blood vessels and the lung, has also been confirmed *in vivo*.

##### Focus on others

In addition to these means, Chen Y. et al. (2020) demonstrated that *F. nucleatum* might promote tumor metastasis by activating the autophagy pathway and that the autophagy inhibitor, chloroquine (CQ), could reverse this phenomenon *in vivo* and *in vitro*. The reason for this observation was that *F. nucleatum* infection upregulated the expression of CARD3, a protein thought to be involved in bacterial infection-related autophagy, and activated autophagy then promoted migration. Moreover, this study also showed that *F. nucleatum* might promote metastasis by regulating epithelial-mesenchymal transition (EMT), though there was no in-depth exploration of the mechanism involved. In another study, researchers suggested that *F. nucleatum* promoted EMT by altering cancer cell metabolism. Mechanistically, *F. nucleatum* can active the TLR4/Keap1/NRF2 pathway then up-regulates CYP2J2, a cytochrome P450. Then 12,13-EPOME, a metabolite of CYP2J2, can activate EMT *in vitro* and promote metastasis of CRC *in vivo* (Kong et al., 2021).

#### Chemoresistance

After radical surgery, CRC patients usually receive combination chemotherapy, including oxaliplatin, 5-fluorouracil (5-FU), and others (Chiorean et al., 2020). However, some patients are not sensitive to chemotherapeutic drugs, which eventually leads to tumor recurrence. Previous studies have proven that the gut microbiota plays a positive or negative role in antitumor therapy (Helmink et al., 2019), so intratumor bacteria may be the cause of CRC chemoresistance. Some studies have focused on the impact of *F. nucleatum* on the chemoresistance of CRC. Zhang et al. (2019) suggested that *F. nucleatum* upregulated the expression of BIRC3 via the TLR4/NF-κB pathway in CRC cells. Through BIRC3, a protein that can directly inhibit the caspase cascade and reduce cell apoptosis, *F. nucleatum* reduced the responsiveness of CRC cells to 5-FU. Moreover, Yu et al. (2017) suggested that *F. nucleatum* induced the resistance of CRC cells to oxaliplatin and 5-FU by selectively losing miR-18a^∗^ and miR-4802, then activating the autophagy pathway and preventing cells from posttreatment apoptosis, and finally promoting recurrence. Although *F. nucleatum* had been founded to contribute to CRC chemoresistance by inducing cancer cell autophagy and apoptosis, the exact virulence factors or metabolites of *F. nucleatum* responsible for this have not been identified, and the mechanisms during this process still need further investigation.

### *Fusobacterium nucleatum* Reprograms the TME

The TME consists of immune-inflammatory cells, stromal cells, secreted products, metabolites, and the extracellular matrix (Maman and Witz, 2018). In addition, recent studies have proven that the existence of microbiota in various tumor tissues is not special but a common phenomenon (Nejman et al., 2020). Therefore, the intratumor microbiota is also considered a part of the TME. The TME plays a vital role in the development of CRC; for instance, it impacts tumors through angiogenesis and immune regulation. Moreover, there are complex interactions between the TME and cancer cells (Maman and Witz, 2018).

#### *Fusobacterium nucleatum* Interacts With Cancer Cells

The development of CRC is often accompanied by inflammation (Schmitt and Greten, 2021). Inflammatory cells and inflammatory cytokines have been considered active tumor factors since they can induce angiogenesis, and stimulate cancer cells proliferation and invasion (West et al., 2015; Galdiero et al., 2018). In addition to inflammatory cells, cancer cells stimulated by *F. nucleatum* can also produce a variety of inflammatory cytokines, such as IL-6, IL-8, and TNF-α, which are related to activation of the NF-κB pathway (Kostic et al., 2013; Rubinstein Mara et al., 2013; Koi et al., 2018). These inflammatory cytokines can act on cells that express their specific receptors in the TME and promote tumor progression in a variety of ways.

In addition to inflammatory cytokines, cancer cells infected with *F. nucleatum* can secrete chemokines. As mentioned earlier, IL-8 and CXCL1 not only stimulated the migration of noninfected HCT116 cells but also recruited immune cells, particularly neutrophils, to the tumor site and increased inflammation (Casasanta et al., 2020). Moreover, *F. nucleatum* stimulates cancer cells to produce CCL-20. Once CCL-20 binds to its ligand CCR6, it can recruit CCR6^+^ immune cells, including subsets of IL17-expressing T helper cells (Th17), regulatory T cells, and dendritic cells (Ye et al., 2017).

Tumor-derived exosomes (TEXs) are important components of the TME, as they can deliver ncRNAs and proteins to recipient cells, which contribute to intercellular communication and molecular transfer (Xu et al., 2018). In addition to being internalized by uninfected cancer cells mentioned above, they may also be internalized by noncancerous cells to reprogram the TME, though Guo S. et al. (2020) did not show this phenomenon. They can even enter body fluids or the blood, mediating remote regulation between organs (Xu et al., 2018).

The success of metastasis depends not only on the metastatic capacity of cancer cells but also on the assistance of the TME (Hinshaw and Shevde, 2019). For instance, the initial stage of local tumor spread requires hydrolysis of the extracellular matrix to overcome the tissue barrier. Some researches have shown that in breast and oral cancers, *F. nucleatum* can induce the secretion of MMP9, which can degrade basement membrane collagen and provide conditions for metastasis (Gholizadeh et al., 2016; Gonzalez-Avila et al., 2019; Parhi et al., 2020). However, this phenomenon has not been reported in CRC.

#### *Fusobacterium nucleatum* Interacts With Immune Cells

The most abundant cells in the TME are infiltrating immune cells and mesenchymal support cells (Maman and Witz, 2018). Though the interaction between *F. nucleatum* and mesenchymal cells, such as cancer-associated fibroblasts, has rarely been studied in CRC, the modulation of the tumor-immune microenvironment by *F. nucleatum* is considered to be an essential component of its tumorigenesis. There are two types of tumor-infiltrating immune cells: antitumor cells, such as natural killer cells (NK cells) and cytotoxic CD8^+^ T cells, and tumor-permissive immunosuppressive cells, such as myeloid-derived stem cells (MDSCs) and tumor-associated macrophages (TAMs). Overall, *F. nucleatum* reshapes the tumor-immune microenvironment and accelerates CRC progression by increasing the number and function of immunosuppressive cells and inhibiting antitumor cells.

Kostic et al. (2013) provided compelling evidence that *F. nucleatum* selectively expanded MDSCs, TAMs, tumor-associated neutrophils (TANs), and dendritic cells (DCs) in APC *^Min+^* mouse models. These cells not only suppress immunity but also participate in the biological behaviors of tumors. For instance, M2-like TAMs not only inhibit T cells through the expression of arginase-1 (Arlauckas et al., 2018), but also promote proliferation, metastasis, and angiogenesis by secreting numerous growth factors and chemokines (Galdiero et al., 2018). Researchers attributed this selection expansion to the metabolites of *F. nucleatum*, such as SCFAs and formyl-methionyl-leucyl-phenylalanine, which act as chemoattractants of MDSCs (Kostic et al., 2013), though no experiments were conducted to confirm this hypothesis. Another study also showed that *F. nucleatum* promoted the M2 polarization of macrophages via the TLR4/IL-6/p-STAT3/c-MYC cascade (Chen et al., 2018).

Epidemiological studies have revealed that a high abundance of *F. nucleatum* is associated with a low level of CD3^+^ T cells (Mima et al., 2015), specifically CD3^+^ CD4^+^ CD45RO^+^ cells (Borowsky et al., 2021). This may be because *F. nucleatum* has a direct or indirect inhibitory effect on CD3^+^ T cells. Kaplan et al. (2010) demonstrated that *F. nucleatum* used its outer membrane proteins Fap2 and RadD to induce the death of Jurkat cells, human lymphocytes, in a manner that doesn’t depend on the complete bacterial structure (Kaplan et al., 2010). In addition, Fap2 binds to TIGIT receptors on NK cells and other tumor-infiltrating T cells. TIGIT can inhibit immune cytotoxicity and protect *F. nucleatum* and cancer cells from being killed (Gur et al., 2015).

#### *Fusobacterium nucleatum* Interacts With BMSCs

Bone mesenchymal stem cells (BMSCs), pluripotent stem cells with the abilities to self-renew and differentiate, are involved in the development of CRC (Koliaraki et al., 2017). Lin et al. (2020) explored the effect of BMSCs on *F. nucleatum*-induced CRC. The authors verified that *F. nucleatum* induced BMSCs to transplant to the submucosa and mucosal layer and simultaneously activated the Wnt signaling pathway. Furthermore, after stimulation with *F. nucleatum*, BMSCs accelerated the tumorigenesis of CRC by increasing the secretion of the Wnt3a protein. In summary, *F. nucleatum* modulates the TME to accelerate tumorigenesis by BMSCs.

## Directions for Future Research

Although extensive studies have deeply explored the relationship between *F. nucleatum* and CRC, there is still much unresolved. First, it will make sense to find a more convenient method for patients using *F. nucleatum* as a biomarker for CRC. Considering that patients with *F. nucleatum* bacteremia were at high risk of CRC (Kwong et al., 2018), whether the blood test for *F. nucleatum* DNA alone could be developed as an early screening technique for CRC is still an open question. Second, FadA, Fap2, and LPS, which have been widely accepted to contribute to the pathogenicity of *F. nucleatum*, that is FadA and Fap2 enable *F. nucleatum* to adhere to cells and LPS regulate the inflammation signal through the innate immune pathway, so is there any other specific cell components or metabolites of *F. nucleatum* realize its pathogenetic role still need further investigation (Zanzoni et al., 2017; Cochrane et al., 2020). In addition, whether *F. nucleatum* would affect other important phenomena in the entire TME is also worth exploring, such as abnormal metabolism, tumor angiogenesis, and hardness of the tumor matrix (Nia et al., 2020). Emerging research techniques, like multi-omics joint analysis, single bacteria/cell sequencing technology may help solve these problems. Third, experimental results should be interpreted more carefully, because we cannot define *F. nucleatum* as totally a harmful or a pro-carcinogenetic bacterium. The role of *F. nucleatum* to CRC depends on the genetic background of the host, the heterogeneity of the tumor, the associated TME, and other environmental factors. So, the traditional *in vitro* or *in vivo* experiments hardly take into consideration of these confounding factors. More ingenious or specific experiments should be carried out, such as organoid (Lau et al., 2020), PDX model (Invrea et al., 2020), and organ-on-a-chip (Del Piccolo et al., 2021; Puschhof et al., 2021), which somehow gives the results closer to the real world. Fourth, chemotherapy/immunotherapy resistance is still a tough problem for the oncologist, and few studies claim that *F. nucleatum* leads to chemoresistance by regulating autophagy or apoptosis (Yu et al., 2017; Zhang et al., 2019), but the exact mechanism is still needed to be figured out. The effects of *F. nucleatum* on pharmacotherapy come down to three aspects. Firstly, *F. nucleatum* regulates the cancer cells directly to equip them with resistance. *F. nucleatum* could also affect the metabolite process of pharmaceutical molecules directly or indirectly in bodies. Finally, the therapeutic effects no doubt depends on the TME (Binnewies et al., 2018), so *F. nucleatum* could regulate the components of TME to realize the resistance to therapy.

In summary, precisely defining mechanisms by which *F. nucleatum* is involved in the progression, metastasis, and treatment response of CRC will potentially become the cornerstone of the establishment of CRC management methods based on *F. nucleatum* in the future.

## Author Contributions

SW and YW conceived the review. SW and YL searched the literature and drafted the manuscript. JL and LZ polished the language. WY drew the figure. BL and XG made the tables. YW edited the manuscript. All authors read and approved its final version.

## Conflict of Interest

The authors declare that the research was conducted in the absence of any commercial or financial relationships that could be construed as a potential conflict of interest.

## Publisher’s Note

All claims expressed in this article are solely those of the authors and do not necessarily represent those of their affiliated organizations, or those of the publisher, the editors and the reviewers. Any product that may be evaluated in this article, or claim that may be made by its manufacturer, is not guaranteed or endorsed by the publisher.
